# A Case of Impacted Head

**Published:** 1853

**Authors:** E. G. Harris

**Affiliations:** Fayette, Ala.


ARTICLE IV.—*A Case of Impacted Head.*—By E. G HARRIS, M. D., of Fayette, Ala.

Mrs. Jane R * * * * * * * is a lady about 22 years of age, fair constitution, and since her marriage, which was in the spring of 1852, has enjoyed fine health. From the time her catamenia made their appearance, until her 20th year, she had several attacks of epilepsy.—If the menstrual flux failed to make its appearance at the proper time, the nervous system never failed to make it known by a violent convulsion which came on without any premonitions, and lasted from ten to thirty minutes, leaving her drowsy and inactive, both physically and mentally. This, however, gradually wore off in two or three days, and she would feel as well as usual.—Her menses generally made their appearance soon after the convulsion, though scanty, and would cease too soon—when ever the uterine secretion was free she had no convulsion.—In her 20th year, I was called on to prescribe for her—I felt confident that could 1 establish a free flow of the catamenia at their proper time, and give sufficient tone and strength to the nervous system, to carry on this function without the aid of medicine, that this was all that was necessary to effect a cure of the convulsions. I accordingly gave one grain of the dried Sulph. Feri, with two of Myrrh, three times a-day, commencing two weeks before her expected monthly period, and continued until within two days of that time, then to take a pill composed of two grains of soct. Aloes, with two of Gum Guiac., three times a-day, at the same time to take pretty freely of a decoction of Rad. Senega, warm pediluvia at night, and if there was any uneasy sensations in the uterine region, with lumbar pains, to use the warm hip-bath. This treatment was to be kept up until the flow was free, and then stopped. After carrying out the above treatment for some twelve months, and having no return of the convulsions, it was thought unnecessary to continue it longer. Some six months now elapsed, and her health being as good as could be desired, she married. Soon after her marriage she became pregnant, and during her time of utero gestation she enjoyed excellent health, becoming very corpulent, and perfectly free from those nervous symptoms incident to women in this condition.

Early in the night of the 2d of September last, she was taken in labor.—an old lady who was the midwife for the neighborhood was sent for, and after working hard until her strength was exhausted and her "larnin" baffled, a messenger was dispatched for me. On my arrival, about daylight, I found the pains hard and frequent, the head low down in the inferior strait, and in a transverse position, one ear under the os Pubis and the other to the Sacrum—the anterior fontenelle presented. The os uteri was sufficiently dilated, but there was considerable rigidity in the external parts. I placed two fingers upon the upper portion of the os frontis, and in the absence of pain, used considerable force in order to throw the chin upon the superior portion of the sternum, but to no effect. The manipulations used previous to my arrival had produced considerable tumefaction in the soft parts, with great heat and tenderness. I now applied ointment of belladona freely, and, after waiting a proper time, gave her bountifully of Uva Ursi tea. This increased the uterine contractions to such a degree, that I was indeed afraid the uterus would rupture, though the head of the child moved not an hair's breadth. I now gave her half a grain of Morphine, hoping to still the violent contractions of the uterus, and sent after my forceps—at the ssme time I sent a note to Dr. S. B. Abernathy, requesting his assistance. I should have stated, in connection with the use of the Belladona Ointment, which 1 applied several times, also warm water was repeatedly used, both by bathing and injection, but all to no effect. The Morphine had little or no effect in lessening the contractions. She continued to suffer. 1 attempted the administration of Chloroform, but owing partly to her fear, but mostly to the violent opposition of her husband, we were compelled to cease its use, without doing much good. Dr. Abernathy arrived about noon with the forceps—by this time considerable fever had come on, pulse 130, with great thirst. I should have stated that when I sent for my forceps I also sent for my lancet, believing she would have to be bled. We were satisfied the child was dead, but how long this had been the case we knew not. On consultation with Dr. Abernathy, we determined to bleed her until an impression was made on the pulse. We then raised her to a semi-recumbent position, and took 30 ounces of blood, when she became somewhat faint and turned pale. No visible effect was produced except lessening the uterine contractions. We then gave her some nourishment, and concluded to wait a while and see if the system would not recover its lost tone, and when the pains commenced again to increase their force by Uva Ursi or Ergot. She remained in this condition until about ten o'clock at night, with an occasional pain but not suffering much, except from pressure on the soft parts. We then attempted to increase her pains by the above-mentioned teas—used Belladona Ointment to the vagina and vulva freely and frequently, also warm water bountifully, changed her position from her side to her back, and from a recumbent to a standing position, but all our efforts were in vain, the contractions were increased but slightly, the pulse rapid, skin hot, thirst great, and considerable prostration. The night passed off in fruitless attempts to relieve her, without a resort to the forceps. We were satisfied something must be done. She had already suffered more than mortal tongue could tell. Prostration was coming on very fast, and, unless speedily relieved, death would be inevitable. Not the least advance had taken place in the head. We feared some deformity of the shoulders, or the head would have been opened, so, by day-light in the morning we prepared to use the forceps. We accordingly placed her across the bed, her nates projecting some two or three inches over the bed-rail, and her feet separated the proper distance, rested on a couple of chairs. I then introduced the right blade of Dewees forceps without much difficulty or pain to the patient. I then introduced the other, but with more difficulty, as I was very cautious and desisted whenever she complained much· Not more than five minutes elapsed in the introduction of both blades. I now locked them, and as there were yet some pains, every fifteen or twenty minutes, I commenced gentle traction with the pain, and ceased as they went off. This was kept up for near an hour, and nothing effected—the amount of traction was then increased at each pain for more than an hour—by this time nearly all the strength I had was spent at each attempt—a gentle rotary motion was kept up all the time. It was now plain to be seen that the head was advancing. This encouraged us to continue our efforts, which was done for some three hours, when the delivery was effected. As the child merged out from under the pubis, some thirty or forty ounces of very fœtid Liquor Amnii, which had been pent up and could not pass the impacted head, made their escape, and were so very offensive that it was with great difficulty the assistants and physicians could stay in the house or suppress vomiting. The child looked like it might have been dead several days—it was of medium size, rather large shoulders and chest, abdomen much swollen and covered with livid spots. The placenta soon followed, with some hemorrhage and considerable prostration. Stimulants, however, soon rallied her, and she took some nourishment. I visited her daily—during the first week I gave her tonic doses of Quinine three times a-day, a mild aperient every twenty-four or thirty-six hours, washes and vaginal injections of tepid milk and water, also a sol. Chloride of lime was used daily to destroy the fœtor. There was some sloughing of the soft parts, but not as much I expected. Her urine was drawn with the catheter for some days, after this it passed off as it was secreted, without her having any control over it for a week. She now began to improve, and very gradually recovered. At this time, she is nearly as well as ever, and can very well attend to her domestic affairs. It is very evident that the head was not impacted from its size, but partly from its position, and partly from contracted pelvis—the face presented to the mother's right thigh and the occiput to the left. Mrs. R., with all her connections, seems to be of rather a strumous diathesis. She has a sister who has had curvature of the spine for a number of years. She has three sisters married, all of whom suffer greatly in their confinements.

Nov. 9th, 1853.

